# Impact of public health education on the health status of the older migrant population

**DOI:** 10.3389/fpubh.2022.993534

**Published:** 2022-09-13

**Authors:** Zaohong Yan, Fang Han, Runguo Gao, Qi Jing, Qianqian Gao, Weiqin Cai

**Affiliations:** ^1^Intensive Care Unit, Affiliated Hospital of Weifang Medical University, Weifang, China; ^2^Department of Pathology, Affiliated Hospital of Weifang Medical University, Weifang, China; ^3^School of Public Health, Weifang Medical University, Weifang, China; ^4^School of Management, Weifang Medical University, Weifang, China

**Keywords:** population aging, public health, health education, influential factors, migrant population

## Abstract

**Background:**

Due to an increasing aging population, China has experienced a rapid expansion in its internal older migrant population who face greater health risks and who have a relatively high demand for health education. Public health education is an important means of preventing diseases and promoting health. However, many studies have focused on the utilization, with few studies examining the impact of public health education on the health of the older migrant population in China.

**Objectives:**

This study analyzed the impact of public health education on the health of the older migrant population in China.

**Methods:**

Based on data obtained from the National Migrant Population Health and Family Planning Dynamic Monitor Survey (2018), logistic models and propensity score matching were used to analyze the impact of public health education on the health of the older migrant population.

**Results:**

The self-assessed health of China's older migrant population was good, and the acceptance rate of public health education was 40.81%. The public health status of the older migrant population receiving public health education significantly improved (p < 0.05). After correcting for endogeneity among the variables, public health education increased the probability of improving the older migrant population's self-assessed health by 5.4–6.1% (*p* < 0.01). Heterogeneity analysis found that public health education had a greater impact on the health of older men with an education level of middle school and below, and especially on the older migrant population in the eastern region of China.

**Conclusions:**

Public health education positively affected the health status of the older migrant population. The characteristics and preferences of the older migrant population involving different genders, regions, and educational levels need to be considered, public health education needs to be standardized, and appropriate education methods need to be adopted that suit the older migrant population. Reference suggestions are provided for improving the health level of the older migrant population.

## Introduction

In China, a “migrant population” refers to adults of reproductive age who leave their hometown where their household registration is located and live in different places for the purpose of work and life. This phenomenon is also called population flow. With the rise in population aging in China and the comprehensive introduction of the two-child policy, more older people will enter the migrant population for reasons such as family relocation, reunion, and employment (1). The “China Migrant Population Development Report 2018” found that the older migrant population in China had continued to increase and that, by 2015, the older migrant population had reached 5.03 million, with an annual growth rate of 6.6% (2).

In addition to the dual challenges of “old age” and being a “migrant” (3), the older migrant population faces greater health risks. Relevant literature shows that mobility affects the health status of migrants. The older migrant population has a relatively high demand for health education (4), which highlights their vulnerability. Thus, determining how to ensure the quality of life of the older migrant population and improve their health level is an issue worthy of attention. Public health education is an important means of preventing diseases and promoting health, and it is a world-recognized strategic measure of health care (5). Effective public health education is conducive to enhancing the self-health awareness of older people, improving their health-related behaviors, and establishing a correct concept of health (6). However, for the older migrant population, inadequate economic support and educational level, physiological function decline, and cognitive decline have led to insufficient provision and utilization of public health education. Studies have shown that nearly one-third of the migrant population has not received any public health education (7).

Relevant studies have mainly focused on the working-age migrant population, with few studies undertaken on the older migrant population (8–12). Previous studies have focused on the health of the migrant population in terms of three aspects: the migrant population's health status and influence factor, health knowledge level, and receipt of health education (13–16). Most studies have focused on the utilization of public health services, such as visiting doctors, physical examinations, and hospitalizations, but with research on this population's public health education being relatively limited.

Thus, based on the China's National Health Commission's National Migrant Population Health and Family Planning Dynamic Monitor Survey (2018), this study empirically analyzed the impact of receiving public health education on the health of the older migrant population and assessed differences between various groups. The goal was to provide a reference to improve public health education and the health status of the older migrant population.

## Materials and methods

### Data sources

Data on the older migrant population were obtained from personal questionnaires used in relation to the migrant population in the National Migrant Population Health and Family Planning Dynamic Monitor Survey (2018). The data were based on the sampling frame of the 2017 annual report database of the total migrant population in China's 31 provinces and of the Xinjiang Production and Construction Corps (An important part of Xinjiang Uygur Autonomous Region). Probability Proportionate to Size Sampling (PPS) was adopted. The personal questionnaire mainly included information on: (1) family members, and income and expenditure; (2) employment situation; and (3) use of health and public services.

The older migrant population are defined as individuals who have lived away from the town or street of their household residence for more than half a year, excluding those living in a different town/district in the same city as their household residence. After screening the data, this study included a total of 5,589 migrant older people, all of whom were 60-years old and above.

### Variable selection and description

Self-assessed health status has been reported to be consistent with the actual health level of the individual (17, 18). The explained variable in this study was the self-assessed health status of the older migrant population. The two items “healthy” and “basically healthy” in the questionnaire were combined into one item “relatively healthy,” and the two items “unhealthy, but able to take care of oneself” and “not able to take care of oneself” were combined into one item “less healthy.” The core explanatory variable was the migrant population receiving public health education, as assessed through the questionnaire in terms of “in the past year, whether the migrant population has received the seven items of public health education, including occupational disease prevention and control, reproductive health and women's and children's health, infectious disease prevention and control, chronic disease prevention, prevention and treatment of chronic diseases, mental health, and others.” If the older migrant population had received public health education at least twice, their status of receiving public health education was considered good whereas if they had received it less than twice a year, their receiving status was considered poor. We also considered personal characteristics like gender, age, ethnicity, and education level; family characteristics like average monthly income, duration of migration, scope of migration, and number of children; and participation in medical insurance and establishment of health records. These characteristics and conditions were included in the regression model as control variables affecting health status (19, 20).

### Statistical processing

SPSS 25.0 software was used for statistical analysis. The situation of health education received by the older migrant population was described using descriptive analysis, and a χ2 test was used to analyze differences in health education in relation to different population characteristics. Using binary logistic regression, the indicators with statistically significant differences in the univariate analysis were included as independent variables in the regression model. The effects of health education on health were further assessed using a robustness test and heterogeneity analysis. Propensity score matching (PSM) was used to further verify the impact of public health education on the health of the older migrant population. The test level was two-sided α = 0.05.

## Results

### Health education received by older migrant population with different characteristics

In total, there were 5,589 older migrants included in this study. The surveyed older migrant population included 3,205 men (57.34%) and 2,384 women (42.66%). The majority were 60–69-years old, accounting for 81.36%. Their education level was mainly primary school and below (45.89%) and junior high school (30.33%). Their marital status was mainly married (85.28%), and the average monthly household income was below 3,500 yuan (38.53%). The range of this population included 1,135 people (20.31%) within cities and across counties, 1,946 people (34.82%) within provinces and across cities, and 2,508 people (40.87%) across provinces. The duration of the flow was mainly ≤ 5 years, accounting for 40.46%. There were 1,810 local people (32.39%) with established health records, 3,228 people (57.76%) without established health records, and 551 people (9.86%) who did not know about their health records. Further, 94.04% of the older migrants had at least one medical insurance. Finally, 66.56% had two or more children. Table 1 presents the results.

**Table 1:** Health education received by the older migrant population with different characteristics (*N* = 5,589).

**Variable heading**	**Number of people surveyed**	**Number of people receiving health education (more than twice)**	**Acceptance rate**	**χ^2^ value**	**p-value**
**Gender**
Men	3,205	1,360	42.43%	8.177	0.004
Women	2,384	921	38.63%		
**Age (years)**		
60–69	4,547	1,872	41.17%	3.033	0.219
70–79	915	366	40.00%		
≥80	127	43	33.86%		
**Marital status**		
Single	823	189	22.96%	0.007	0.932
Not single	4,766	2,092	43.89%		
**Nationality**		
Han nationality	5,138	1,847	35.95%	0.243	0.622
Other nationalities	451	161	35.70%		
**Educational level**		
Elementary school and below	2,565	1,001	39.03%	8.790	0.032
Junior high school	1,695	731	43.13%		
High school/secondary school	922	391	42.41%		
University and above (including specialist)	407	158	38.82%		
**Average monthly household income (yuan)**		
3,500 and below	2,034	819	40.27%	13.271	0.004
3,500–6,000	1,426	634	44.46%		
6,000–10,000	1,202	482	40.10%		
10,000 and above	924	344	37.23%		
**Duration of migration**		
5 years and below	2,261	917	40.56%	10.404	0.006
5–10 years	1,495	658	44.01%		
10 years and above	1,833	706	38.52%		
**Reasons of migration**		
Work and business	2,271	642	28.27%	8.156	0.017
Care for children, grandchildren	1,324	1,212	91.54%		
Pension or other	1,994	427	21.41%		
**Range of migration**		
Cross province	2,508	889	35.45%	56.723	0.000
Cross city	1,946	900	46.25%		
Within city	1,135	492	43.35%		
**Number of children**			
1 child	1,869	799	42.75%	4.365	0.037
2 or more	3,720	1,483	39.87%		
**Health record**		
Yes	1,810	1,073	59.28%	384.988	0.000
No	3,228	1,060	32.84%		
Unclear	551	148	26.86%		
**Medical insurance**		
At least one	5,256	2,716	51.67%	12.626	0.000
None or not clear	333	105	31.53%		
**Family doctor contract**	
Yes	983	644	65.51%	301.290	0.000
No or unclear	4,606	1,637	35.54%		

### Situation of the older migrant population with different characteristics receiving public health education

From the perspective of health status, 82% of the older migrant population believed their health status was good and basically healthy. In terms of receiving public health education, 40.81% of the older migrant population had received public health education at least twice. The analysis found that the acceptance rate among the older migrant population of public health education differed by gender, education level, average monthly family income, duration of migration, reasons of migration, range of migration, number of children, establishment of health records, and medical insurance (*p* < 0.05). Table 1 presents the detailed results.

### Analysis of the impact of receiving public health education on the health of the older migrant population

#### Logistic regression analysis of the influence factor of health status of the older migrant population

When controlling for regional fixed effects, the single variable of receiving public health education added to model (1) was found to have a significant positive correlation with the health status of the migrant population. In model (2), individual characteristic control variables were added, and it was found that receiving public health education significantly improved the health level of the migrant population (β = 0.455). In model (3), family characteristic variables were further added and similar results obtained. In model (4), all the control variables were added, and it was shown that the migrant population receiving public health education had significantly improved health levels (β = 0.482). Therefore, public health education of older migrant groups can promote the public health knowledge of these groups to a certain extent and, at the same time, improve health awareness, which is helpful for disease prevention and improvement of health status.

In terms of personal characteristics, men's health status was better than that of women's health status. The migrant population with higher education levels also showed a corresponding increase in health awareness. The regression results showed that health status was positively correlated with education level. In terms of family characteristics, groups with higher family income had a relatively high overall quality of life, and a close family life also had a positive effect on health and wellbeing. The range and duration of the flow were negatively correlated with health status, suggesting that a long-term flow affects the health status of older people. The number of children was negatively correlated with health status, which might be explained in terms of some migrants needing to spend more time and energy in taking care of their children due to their long-term separation from their children, which would likely affect their psychological status and health. Table 2 presents the results.

**Table 2:** Impact of receiving public health education on the health of the older migrant population.

**Variable**	**Self-assessed health status**			
	**Model (1)**	**Model (2)**	**Model (3)**	**Model (4)**
Receipt of public health education	0.385**	0.455*	0.444**	0.482**
	(0.073)	(0.076)	(0.077)	0.081
Gender		−0.372**	−0.353**	−0.349**
		0.072	(0.075)	0.075
Education level		0.431**	0.299**	0.302**
		(0.046)	(0.049)	0.050
Average monthly household income (yuan)			0.353**	0.349**
			(0.039)	0.039
Duration of migration (years)			−0.216**	−0.209**
			(0.043)	0.043
Reasons of migration			0.300**	−0.296**
			(0.056)	0.056
Range of migration			−0.118**	−0.120**
			0.048	0.049
Number of children			−0.472**	−0.473**
			0.089	0.089
Whether participated in medical insurance				0.141**
				0.151
Whether established health record locally				0.080
				0.069
Whether family doctor service contracted				−0.078
				0.109
Regional fixed effects	Control	Control	Control	Control
Constant term	1.370**	1.687**	3.012**	2.707**
Sample size	5,589	5,589	5,589	5,589
Hosmer-Lemeshow *p*-value	0.000	0.150	0.181	0.718
*R* ^2^	0.008	0.071	0.125	0.126
Omnibus test	28.424**	248.284**	444.122**	448.257**

*p < 0.05; **p < 0.01. The results reported by all models are β values, standard error in parentheses.

#### Robustness check

To enhance the research conclusions' robustness, the following two methods were used for testing. First, concerning self-assessed health options: “cannot take care of oneself, unhealthy but can take care of oneself, basic health,” a value of 0 was assigned, while for the option “healthy,” a value of 1 was assigned, with binary logistic regression used for analysis. Second, we then replaced the explained variable with “illness (injury) or physical discomfort” as an objective indicator and again used binary logistic regression for analysis. The robustness test results are shown in Table 3.

**Table 3:** Robustness test results.

**Variable**	**Model (1)**	**Model (2)**
	**Self-assessed health**	**Illness (injury) or physical discomfort**
Receive public health education (at least two)	1.386**	1.513**
	0.060	0.066
Other control variables	Control	Control
Regional fixed effects	Control	Control
Sample size	5,589	5,589
Likelihood-ratio statistics	7,316.349**	6,533.181**
Pseudo *R*^2^	0.092	0.059
Hosmer-Lemeshow *p*-value	0.737	0.613
Omnibus test	399.960**	236.641**

**p < 0.01. Binary Logistic regression was used for model (1) and model (2). All models reported results as OR values.

In model (1), we found that in the condition of receiving public health education, the probability of the older migrant population being healthy was significantly improved, with the probability of an increase in health status being 0.386. In model (2), we found that the probability of improving the health of the older migrant population increased by 0.513 under the condition of receiving public health education. The above two test methods showed that the models were robust.

#### Heterogeneity analysis

To explore the heterogeneity of the impact of receiving public health education on the health status of different groups, this study grouped the older migrant population according to gender, region, and educational level, as shown in Table 4. Models (1) and (2) showed that receiving public health education significantly improved the health status of both older men and women, and its impact on older women was greater than that on older men. Models (3) and (4) showed that receiving public health education had the greatest impact on the health status of the older migrant population in eastern regions of China. Models (5) and (6) showed that public health education affected the health status of the older migrant population differently depending on educational levels, with a significant effect on those with a low level of education.

**Table 4:** Heterogeneity test results.

**Variable**	**Gender**		**Region**		**Education level**	
	**Model (1)**	**Model (2)**	**Model (3)**	**Model (4)**	**Model (5)**	**Model (6)**
Subgroup	Male	Female	East area	Midwest	Junior high school and below	High school and above
Public health education	0.364*	0.580**	0.508**	0.445**	0.519**	0.239
Other control variables	Control	Control	Control	Control	Control	Control
Regional fixed effects	Control	Control	–	–	Control	Control
Sample size	3,205	2,384	2,271	3,318	4,260	1,326

**p < 0.01; *p < 0.05.

#### Analysis of endogenous issues

To reduce estimation errors and address potential issues with endogeneity in the analysis, PSM was used to further verify the impact of public health education on the health status. We used nearest neighbor matching and radius matching. Taking the self-assessed health status of the older migrant population as the explained variable and using 1:1 nearest neighbor matching, the standardized deviations of the variables after 1:1 nearest neighbor matching were lower than that before matching, with most being <10%. Compared to the results before matching, the standardized deviations of most variables were greatly reduced, and the matching results were acceptable. Table 5 presents detailed results.

**Table 5:** Variable error reduction.

**Variable**	**Sample**	**Average value**		**Standardized deviation (%)**	**Error reduction (%)**	***t*** **-test**	
		**Test group**	**Control group**			***t*-value**	***p*-value**
Gender	Before matching	1.404	1.442	−7.69%	72.81%	−2.827	0.005
	After matching	1.42	1.41	2.09%		0.651	0.515
Education level	Before matching	1.871	1.839	3.44%	−28.26%	1.267	0.205
	After matching	1.897	1.856	4.42%		1.375	0.169
Duration of migration	Before matching	1.908	1.934	−3.08%	39.13%	−1.136	0.256
	After matching	1.933	1.917	1.88%		0.585	0.559
Reasons of migration	Before matching	1.906	1.902	0.57%	−336.04%	0.208	0.835
	After matching	1.906	1.89	2.48%		0.772	0.44
Range of migration	Before matching	1.827	1.705	15.87%	99.16%	5.84	0
	After matching	1.804	1.805	−0.13%		−0.042	0.967
Household monthly income	Before matching	2.154	2.201	−4.30%	40.67%	−1.585	0.113
	After matching	2.186	2.158	2.55%		0.795	0.427
Number of children	Before matching	1.65	1.676	−5.63%	51.69%	−2.064	0.039
	After matching	1.654	1.667	−2.72%		−0.847	0.397
Participation in health insurance	Before matching	0.954	0.931	9.83%	31.43%	3.676	0
	After matching	0.948	0.962	−6.74%		−2.101	0.036
Establishment of health record	Before matching	1.594	1.899	−51.32%	95.34%	−18.762	0
	After matching	1.694	1.708	−2.39%		−0.745	0.456
Contracted family doctor service	Before matching	0.283	0.103	46.90%	98.25%	16.66	0
	After matching	0.168	0.172	−0.82%		−0.256	0.798

The average treatment effect for the treated (ATT) values are shown in Table 6. Under the two matching methods, the ATT estimation results were 5.4 and 6.1%, respectively, and the *t*-values were 4.812 and 5.128, respectively, both of which were significant at the 5% level. Further, public health education increased the probability of better health status for the older migrant population by 5.4–6.1%, which was consistent with the estimated results of the logistic model.

**Table 6:** Propensity score matching estimation results.

**Matching method**	**Test group**	**Control group**	**ATT effect size**	**Standard error**	***T*-value**	***P*-value**
Nearest neighbor match	0.853	0.799	0.054	0.011	4.812	0
Radius match (0.02)	0.853	0.792	0.061	0.012	5.128	0

ATT, average treatment effect for the treated.

## Discussion

### Low acceptance rate of public health education among the older migrant population

Public health education plays a significant role in popularizing health knowledge and improving health literacy. Due to high mobility and unstable housing, the migrant population faces certain restrictions in receiving public health education, which generates major challenges for effective health education and health care management (21). The results of this study showed that the acceptance rate of public health education for the migrant population was 40.81%, which was lower than the overall acceptance rate of health education for the older migrant population, which was 59.56% (22); this rate was also lower than the utilization rate of public health education for the migrant population. The reason for this finding may be due to a limited health awareness among the older migrant population and that they are busy with their livelihoods and other reasons, as proposed by previous scholars (23). Moreover, the older migrant population are likely to be more affected by a gradual deterioration in their physiological, psychological, and cognitive state due to aging, leading to this population having a low rate of health education. Given the “silver digital divide,” which refers to the phenomenon where older people tend to be less adept at using digital technology and information resources than younger people, it may be that older people do not have equal access to convenient services for information transmission, social interaction, and medical care; they may even be considered excluded (24).

### A significant positive impact of public health education on the health status of the older migrant population

Results of the logistic model analysis in this study showed that when other variables were controlled for, public health education was positively correlated with the health status of the older migrant population. This result held even after addressing endogeneity using PSM. Appropriate public health education is of great significance to ensure the health status of the older migrant population, as it plays an important role in popularizing health knowledge, enhancing health literacy, and advocating healthy lifestyles (25). It can help meet the needs of the older migrant population concerning their own health and also improve their utilization of basic public health services. The older migrant population is affected by multiple factors, such as old age, mobility issues, poor urban adaptability, and poor social support, and their health risks are relatively high. Health literacy is one of the determinants of the health status of the rural older migrant population, and public health education indirectly affects their health status by affecting their health literacy (26). Therefore, it is necessary to create public health education content specifically for the older migrant population in China, fully consider their characteristics, and apply appropriately targeted public health education methods.

### Differences in the impact of public health education on the health status of the older migrant population with different educational levels (the lower the educational level, the greater the impact)

With increasing levels of diversification and urgency in older people's demand for health services (27), the older migrant population has a greater need to maintain their health (28). Public health education is an essential way to maintain and promote the health of older people, especially among those with low education levels. The regression results showed that receiving public health education had a positive correlation with the health status of the older migrant population in relation to different educational levels; however, the effect was greater for those with education level of junior high school and below. The older migrant population generally has a low level of education. Compared with groups with high school education and above, their awareness of self-care in life is relatively weak; therefore, providing public health education is likely to improve their health status. Such public health education should focus on the needs and preferences of the older migrant population, with appropriate targeting given differing educational and cultural levels.

### Greater impact of public health education on the health status of the older migrant population in the eastern region compared with the central and western regions

More resources have been invested in public health education in the eastern region on account of the relatively higher development there of the Chinese economy, and the older migrant population in that region has more choices to choose appropriate high-quality public health education. As the central and western regions are less economically developed, their basic public health service resource investment has been relatively small, and the promotion and implementation of public health education less advanced (29). In addition, as there are still many older migrant people in the central and western regions who have limited health awareness, the utilization rate of public health education in these regions still lags behind that in eastern China. Thus, it is necessary to increase investment in health resources in the central and western regions and take appropriate preferential policy measures. At the same time, a wide range of methods need to be used to promote public health education and improve its utilization rate among the older migrant population in different regions.

## Conclusions

In the context of a rapidly aging population, the older migrant population in China has undergone major expansion. The results of this study showed that public health education plays an important role in improving the health levels of the older migrant population. Greater attention needs to be paid to the positive impact of public health education on the health of the older migrant population. One limitation of this study was that, when selecting the influencing factors of public health education on the health of the older migrant population, this study may have missed other factors that could be of importance. Future research needs to consider a wider range of potentially influential factors when seeking to understand how to improve public health education utilization and health promotion among the older migrant population.

## Data availability statement

Publicly available datasets were analyzed in this study. This data can be found at: https://www.chinaldrk.org.cn/wjw/#/home.

## Ethics statement

Written informed consent was obtained from the individual(s) for the publication of any potentially identifiable images or data included in this article.

## Author contributions

ZY and QG conducted the study design, analysis, and basic writing. QJ and WC drafted some parts of the discussion sections and interpreted the results. RG and FH offered comments to modify the manuscript. All authors read and approved the final manuscript before submission.

## Funding

This research was supported by the National Natural Science Foundation of China (Grant Nos. 72004165, 71673202, and 72104186), the Humanities and Social Science Research Youth Fund Program of the Ministry of Education (Grant No. 20YJCZH002), and the Natural Science Foundation of Shandong Province (Grant Nos. ZR2020QG057 and ZR2021MG019).

## Conflict of interest

The authors declare that the research was conducted in the absence of any commercial or financial relationships that could be construed as a potential conflict of interest.

## Publisher's note

All claims expressed in this article are solely those of the authors and do not necessarily represent those of their affiliated organizations, or those of the publisher, the editors and the reviewers. Any product that may be evaluated in this article, or claim that may be made by its manufacturer, is not guaranteed or endorsed by the publisher.
